# 

**Published:** 1895-08-03

**Authors:** 


					ABSOLUTELY PURE, therefore BEST.
August 3rd, 1895.
CADBURY'S ^ U in CADc? o.RY S
COCOA JiniL JmU? "The Typical Cocoa of English Manufacture -
' r\r i i r ii 1' " ? ^5^555^ Absolutely Pure."?The Analyst*
Of absolute purity and freedom from alkali. ' ?
?Bra.ithwa.iie.
IRIY/CH HOSPI.TAJ.
u^MSCOtr rtsTEHiv Innn??n[ - ^
PRICE TWOPENCE.
xj Xj ? , VT7TTT U/ITH EXTRA NURSING SUPPLEMENT. Per Annum, 10s. 6d? post free.
No. 462: Vol. XYIII. Willi CA III1H nuno Newsraper for Monthly Parts, 6d.; post free, 8d.
QArr,__ , 0/%_ rRetristered at the General Post Office as a IP nif.fnner Annum, 8s.6d., post free*
^ATTTEDAY, August 3rd, 1895. CTanImi!8ion in the United Kingdom and Abroad.]   Dittope

				

